# Commentary: Single-Cell Sequencing Analysis and Weighted Co-Expression Network Analysis Based on Public Databases Identified That TNC Is a Novel Biomarker for Keloid

**DOI:** 10.3389/fimmu.2022.868685

**Published:** 2022-04-04

**Authors:** Yijun Xia, Youbin Wang, Yingjie Xiao

**Affiliations:** ^1^ Department of Plastic Surgery, Peking Union Medical College Hospital, Chinese Academy of Medical Sciences, Peking Union Medical College, Beijing, China; ^2^ Department of Plastic Surgery, Peking Union Medical College Hospital, Beijing, China; ^3^ Department of Cardiothoracic Surgery, Second Affiliated Hospital, School of Medicine, Zhejiang University, Hangzhou, China

**Keywords:** keloid, single-cell sequencing, weighted co-expression network analysis, differential expression analysis, Tenascin-c

## Introduction

Keloids, a common and frequently occurring condition in plastic surgery and dermatology, show tumor-like characteristics such as treatment resistance and a high recurrence rate (1). Keloids are a product of abnormal healing after skin trauma and often cause symptoms such as itching, pain, and local paresthesia within the growth range (2, 3). The diagnosis of keloids generally requires a clinical physical examination after keloid formation. Keloids cannot currently be predicted at the genetic level at an early stage; thus, once keloids are formed in the postoperative incision, the surgical results, especially the surgical results of plastic surgery, will be seriously affected. Therefore, screening and identifying predictive markers of keloids has broad prospects in experimental research and clinical applications of keloids.

We recently read the article by Xie et al. entitled “Single-Cell Sequencing Analysis and Weighted Co-Expression Network Analysis Based on Public Databases Identified That TNC Is a Novel Biomarker for Keloid”, published in December 2021 (4). The authors identified a novel keloid biomarker, Tenascin-c(TNC), by single-cell analysis, weighted co-expression network analysis, and differential gene analysis, and verified TNC expression by RNA-sequencing in clinical keloid samples. These bioinformatic approaches identified a promising therapeutic target and provided a novel diagnostic marker for keloids. However, the mechanism of TNC’s role in the pathological process of keloids has not been fully elucidated. The function and mechanism of TNC in keloids still needs further research, and the process from the identification of keloid biomarkers to clinical translation and clinical application is lengthy.

**Figure 1:**
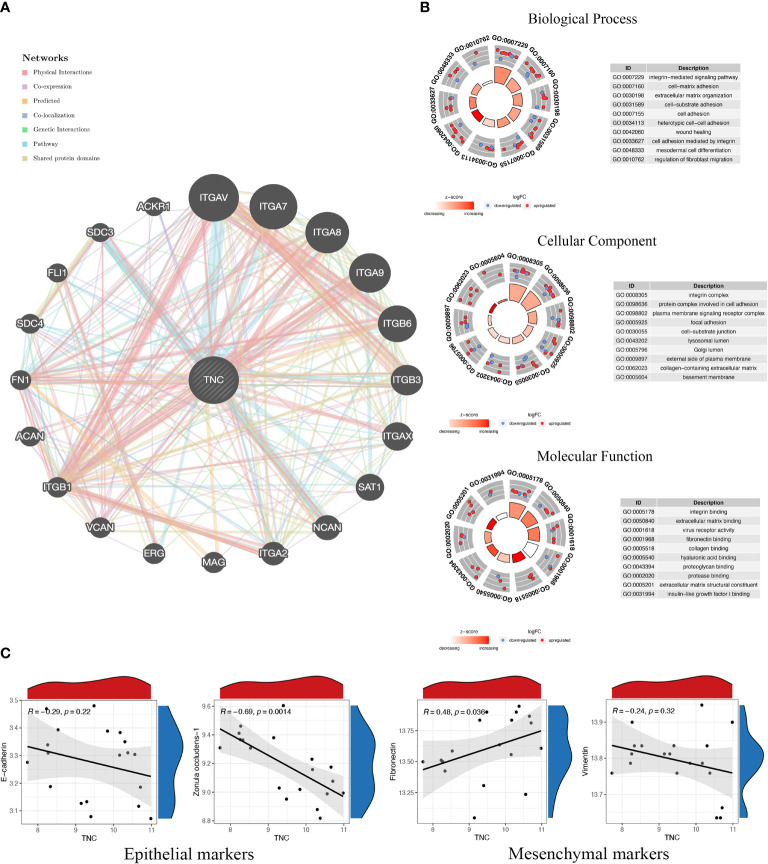
Functional prediction of Tenascin-C and its correlation with markers of epithelial-mesenchymal transition. **(A)** The protein-protein interaction network of Tenascin-C and its related genes was constructed from the Genemania database. **(B)** The functional annotation of Tenascin-C and its related genes was performed in three parts, namely cellular component (CC), molecular function (MF) and biological process (BP). **(C)** The keloid dataset (GSE145725) in the Gene Expression Omnibus (GEO) database was used to perform correlation analysis of Tenascin-C with epithelial markers (E-cadherin and zonula occludens-1) and mesenchymal markers (vimentin and fibronectin).

Based on the article of Xie et al. (4), we made a general prediction of the gene function of TNC in the Genemania database from multiple perspectives including physical interaction, gene co-expression, gene co-localization, and pathway enrichment, and the results showed that TNC mainly interacts with integrin family members (**Figure 1A**). Integrins are a class of cell adhesion factors that participate in cell-to-cell and cell-to-extracellular matrix adhesion and signal transduction by binding to different ligands, and are key regulators of chronic inflammation and fibrosis (5). In the enrichment analysis, TNC and its co-expressed genes were mainly enriched in the extracellular matrix organization, cell adhesion, cell-matrix adhesion, and collagen binding (**Figure 1B** **)**. Notably, the typical pathological features of keloids are the excessive proliferation of fibroblasts and the deposition of extracellular matrix caused by excessive collagen secretion. The functional prediction of TNC is consistent with the pathological mechanism of keloids, so TNC can be regarded as a new signature, which will help elucidate the pathogenesis of keloids from a genetic perspective.

TNC is highly expressed in a tightly regulated spatiotemporal pattern during embryonic development, especially during nervous system development and skeletal formation; however, its expression is negligible in normal mature tissues (6, 7). However, under certain pathological conditions, such as infection, inflammation, trauma and other repair processes, TNC is transiently expressed at the site of tissue remodeling (8). After trauma, the expression of TNC is significantly increased at the wound edge of all layers of the skin, and TNC is present in the entire granulation tissue matrix, filling the full-thickness wound. This increase in expression subsides after wound healing, and if this precise temporal regulation is disrupted, it leads to an imbalance between extracellular matrix synthesis and autophagy that manifests as scarring and keloids (9–11). The protein expression level expression of TNC has been verified in previous studies to be significantly different in normal skin and keloid tissue. The expression of TNC in the epidermis of normal skin and keloid tissue was negative. In the dermis layer of normal skin, TNC expression is characterized by low levels, discontinuity, and a mottled pattern and is restricted to the basal layer and the dermis-epidermal junction. However, in keloids, TNC expression is diffusely distributed in fibroblasts and collagen fibers in the dermis (12, 13).

Tenascin-C can regulate focal adhesion activity, cell migration, extracellular matrix degradation, and epithelial-mesenchymal transition (EMT) processes in a cell-type-specific manner, and this ability to modulate cell behavior is found in various tumor cells and tumor-associated cells. Therefore, TNC is considered to be a key molecule that promotes the formation of a tumor-supportive tissue microenvironment. Notably, the expression of TNC at the invasive edge of tumor tissue is higher than its expression in the cancer nest, indicating that there may be a close relationship between the high expression of TNC and tumor migration (14, 15). Keloids have been classified as fibroproliferative dermal tumors by an increasing number of scholars in recent years, so the effect of TNC on tumor microenvironment construction may have a similar effect in keloids. Immunohistochemical changes in TNC expression have also been used in previous literature to evaluate the efficacy of different keloid treatments. We supplemented the correlation analysis of TNC with epithelial and mesenchymal markers of EMT in the GSE145725 dataset. TNC was negatively correlated with Zonula occludens-1 and significantly positively correlated with fibronectin (**Figure 1C**). However, the role of TNC biosynthesis in keloid fibroblasts in driving processes such as cell adhesion, cell spreading, EMT, and proinflammatory cytokine synthesis has not been fully evaluated experimentally, and this issue is a major limitation of this study.

## Author Contributions

The manuscript was written by YXia with significant contributions from YW and YXiao. All authors contributed to the article and approved the submitted version.

## Funding

This study was supported by The National Natural Science Foundation of China (81871538) and Beijing Municipal Commission of Science and Technology (Z191100006619009).

## Conflict of Interest

The authors declare that the research was conducted in the absence of any commercial or financial relationships that could be construed as a potential conflict of interest.

## Publisher’s Note

All claims expressed in this article are solely those of the authors and do not necessarily represent those of their affiliated organizations, or those of the publisher, the editors and the reviewers. Any product that may be evaluated in this article, or claim that may be made by its manufacturer, is not guaranteed or endorsed by the publisher.
